# OpenFL: the open federated learning library

**DOI:** 10.1088/1361-6560/ac97d9

**Published:** 2022-10-19

**Authors:** Patrick Foley, Micah J Sheller, Brandon Edwards, Sarthak Pati, Walter Riviera, Mansi Sharma, Prakash Narayana Moorthy, Shih-han Wang, Jason Martin, Parsa Mirhaji, Prashant Shah, Spyridon Bakas

**Affiliations:** 1 Intel Corporation, Santa Clara, CA 95052, United States of America; 2 University of Pennsylvania, 3700 Hamilton Walk, Richards Medical Research Laboratories (7th Fl), Philadelphia, PA 19104, United States of America; 3 Albert Einstein College of Medicine, 1300 Morris Park Ave, Bronx, NY 10461, United States of America; 4 Equal first authors

**Keywords:** federated learning, open-source, security, privacy, machine learning, deep learning

## Abstract

**Objective.:**

Federated learning (FL) is a computational paradigm that enables organizations to collaborate on machine learning (ML) and deep learning (DL) projects without sharing sensitive data, such as patient records, financial data, or classified secrets.

**Approach.:**

Open federated learning (OpenFL) framework is an open-source python-based tool for training ML/DL algorithms using the data-private collaborative learning paradigm of FL, irrespective of the use case. OpenFL works with training pipelines built with both TensorFlow and PyTorch, and can be easily extended to other ML and DL frameworks.

**Main results.:**

In this manuscript, we present OpenFL and summarize its motivation and development characteristics, with the intention of facilitating its application to existing ML/DL model training in a production environment. We further provide recommendations to secure a federation using trusted execution environments to ensure explicit model security and integrity, as well as maintain data confidentiality. Finally, we describe the first real-world healthcare federations that use the OpenFL library, and highlight how it can be applied to other non-healthcare use cases.

**Significance.:**

The OpenFL library is designed for real world scalability, trusted execution, and also prioritizes easy migration of centralized ML models into a federated training pipeline. Although OpenFL’s initial use case was in healthcare, it is applicable beyond this domain and is now reaching wider adoption both in research and production settings. The tool is open-sourced at github.com/intel/openfl.

## 1. Introduction

In the last decade, artificial intelligence (AI) has flourished due to greater access to data (Paullada *et al* 2020). Training robust machine learning (ML) and deep learning (DL) models require large quantities of diverse training data to ensure robustness and generalizability to unseen out-of-sample data (Zech et al 2018, Mårtensson et al 2020). However, accessing the requisite amounts of diverse data remains challenging because of various technical (storage, bandwidth), regulatory, or privacy concerns (Sheller et al 2020).

**Figure 1.**
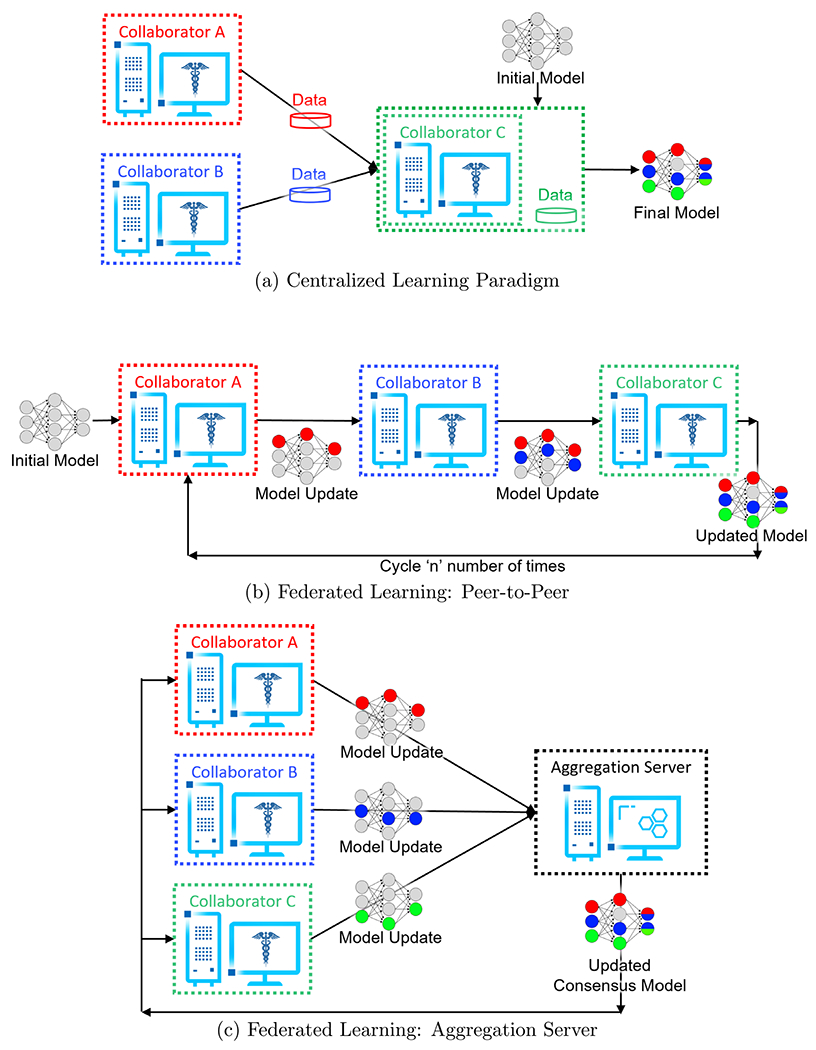
Collaborative learning workflows. (A) indicates the current paradigm for collaborative learning by sharing the local patient data at a centralized location. (B) and (C) show FL approaches for collaborative learning without sharing any local data.

Federated learning (FL) is a computational paradigm that enables organizations to collaborate on ML/DL data science projects, without sharing sensitive information, such as patient records (protected health information), financial transactions, or protected secrets (McMahan et al 2017, Sheller et al 2019, Yang et al 2019, Rieke et al 2020, Sheller et al 2020). The basic premise behind FL is that the AI model moves to meet the data, instead of the data moving to meet the model that represents the current paradigm for multi-site collaborations (figure 1).

Our motivation for this work is two-fold: To lower the barrier for international collaboration, and to enable access to unprecedented and diverse datasets without violating existing privacy laws, such as the Health Insurance Portability and Accountability Act of the United States (Annas et al 2003) and the General Data Protection Regulation of the European Union (Voigt and Von 2017). By achieving this goal, particularly in healthcare applications, FL has the promise to address health disparities, under-served populations, and rare diseases, by gaining knowledge from data coming from institutions that were not able to participate in such collaborative studies before. Literature has shown that ML/DL models trained using FL can achieve comparable levels of performance as models trained using a centralized learning approach (McMahan et al 2017, Sheller et al 2019, Suzumura et al 2019, Sheller et al 2020, Baid et al 2022).

The contribution for this presented work is the open federated learning (OpenFL, github.com/intel/openfl) library introduced here as an open-source, python-based framework for training ML/DL algorithms using the data-private collaborative learning paradigm of FL. Section 2 describes the design and use of OpenFL, with the intention of facilitating its application to existing ML/DL model training in a production environment. Section 2.4 further provides recommendations to secure a federation and how Trusted Execution Environments (TEEs) can ensure explicit model security and integrity, as well as maintain data confidentiality. Section 3 highlights the first real-world applications of the OpenFL library to train consensus ML/DL in the domain of cancer research and beyond. Finally, sections 4 and 5 conclude with some discussion and future directions for the presented work.

## 2. Methods

### 2.1. Synopsis

**Figure 2.**
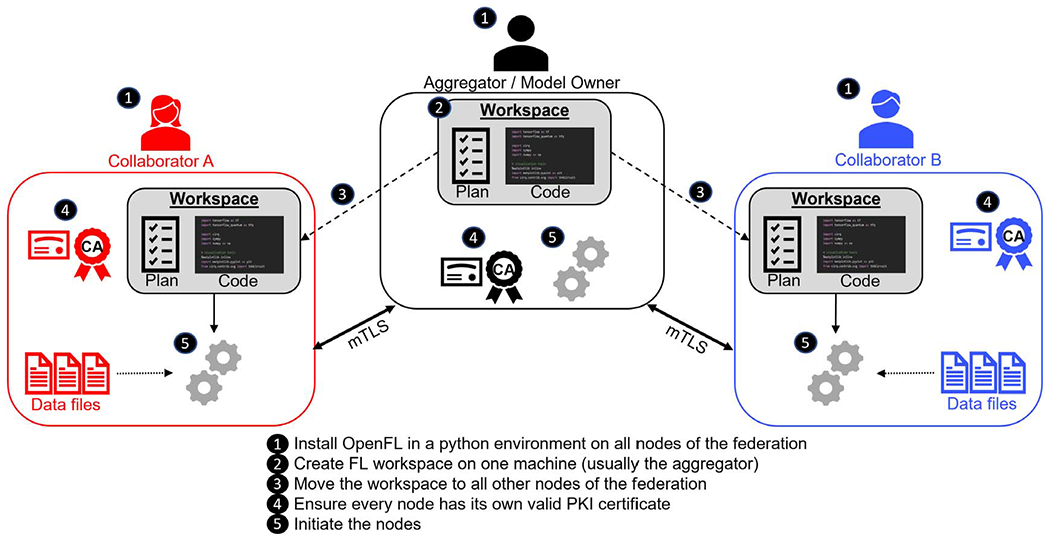
A high-level overview of open federated learning (OpenFL). Note that once OpenFL is installed on all collaborating nodes of the federation and every member of the federation has a valid PKI certificate, all that is needed to run an instance of a federated workload is to distribute the workspace to all federation members and then run the command to start the node.

OpenFL allows developers to train ML models on the nodes of remote data owners (i.e. collaborators). The ML model is trained on the hardware at the collaborator node. The data used to train the model remains at the collaborator node at all times; only the model weight updates and metrics are shared to the model owner. A FL plan is used to describe the configuration and workflow. This FL plan is shared among all nodes in the federation to define the rules of the federation. OpenFL adopts the terminology of FL plan coined by Bonawitz et al (2019), though as OpenFL has been designed for a different trust model (multi-institutional), the OpenFL plan is agreed upon by all parties before the workload begins, as opposed to the design in Bonawitz et al (2019) which delivers the FL plan at runtime (as befits that system’s design goals). The high-level workflow is shown in figure 2. Note that once OpenFL is installed on all nodes of the federation and every member of the federation has a valid PKI certificate, all that is needed to run an instance of a federated workload is to distribute the workspace to all federation members and then run the command to start the node (e.g., fx aggregator start/fx collaborator start). In other words, most of the work is setting up an initial environment (figure 2: steps 1–4) on all of the federation nodes. After the setup, subsequent experiments can be launched quickly after workload redistribution.

### 2.2. Software components

**Figure 3.**
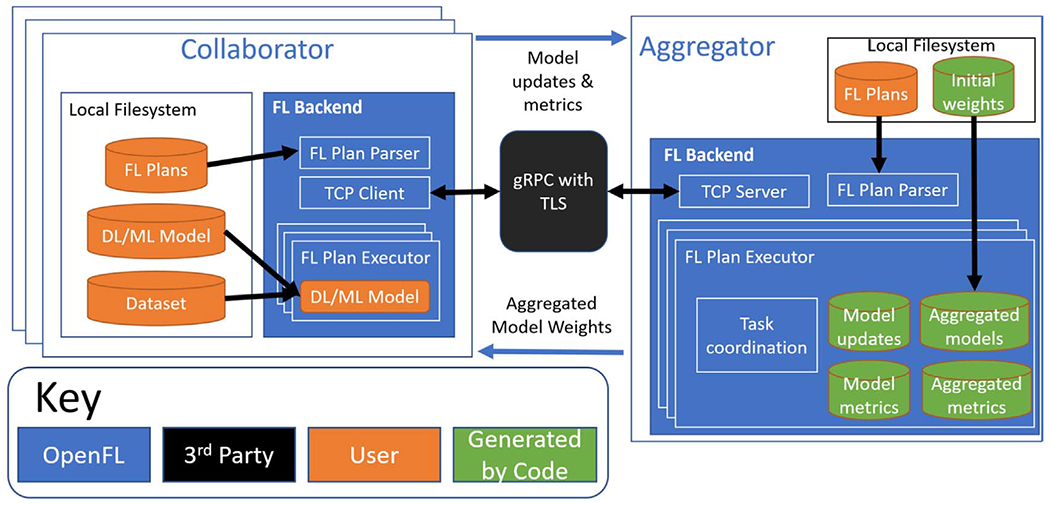
The OpenFL software components. The collaborator contains the federation plan (FL Plan),ML model, and local dataset. These components are created by the developer (orange). The OpenFL backend (blue) connects the collaborator with the aggregator node via a mutual TLS connection. The OpenFL backend (blue) on the aggregator sends remote procedure calls to the collaborator and receives model and metric updates (green) for aggregation.

Figure 3 shows the software components of the OpenFL library. The code is open-source, written in Python, and distributed via pip^5^, conda, and Docker packages.

Every site participating in a collaborative network (*federation*) will need to have information on the predefined federation plan (FL Plan), the ML model code, and the local dataset. The coordination and execution of a given federation is defined by the FL plan. The FL plan is defined within a text file (i.e. YAML^6^), which is shared with all the participants of a given federation. It defines the federation settings, such as batch size, IP address, and rounds to train an AI model. It also specifies the remote procedure calls for the given federation tasks. The FL plan and model code are manually shared with each participant, prior to the initiation of the federation using an export command in the OpenFL command line interface (CLI). A complete description of these steps and commands to execute can be found in the library’s technical documentation^7^.

When the participants start the federation, the OpenFL backend allows the collaborator node to send requests via remote procedure calls to the aggregator to ask which task it should execute next. Tasks are key workload steps, e.g., ML model training and validation, and are defined in the FL plan. In this way, the aggregator can dynamically choose which task to assign to each collaborator, but as the tasks are predefined, cannot send arbitrary commands to the collaborators. Moreover, the dependencies required for the collaborator’s task to execute are sent over the network as numpy arrays, and transformed into the updated model weights immediately before the training or validation function is scheduled to execute. When the collaborators have completed their tasks, collaborators report the updated model weights (and aggregated metrics, such as model accuracy and local dataset size) to the aggregator. The aggregator then combines the updates received from the collaborators into a global consensus model, as described by the algorithm specified in the FL plan. The collaborators then retrieve the weights of the new global consensus model from the aggregator for an additional round of tasks (figure 1). This process continues until all rounds have been completed as specified in the FL plan.

The primary rationale for the design choices in this procedure is to establish trust and maintain security, while making it easier for IT security admins to evaluate the code that will execute within their network. The distribution of both the FL plan and source code gives participants a comprehensive view into the code that can be executed on their machine, and the RPC calls are specifically defined to limit what can be sent across the network. This design lays the foundation for the methods described in sections 2.3 and 2.4, including the use of mTLS for encrypting the network traffic of all parties, and how TEEs can bring hardware-backed code confidentiality and integrity to a federation.

Current examples are artificial neural networks trained using either TensorFlow (Abadi et al 2016), PyTorch (Paszke et al 2019), or MXNet (Chen et al 2015). Other ML model libraries and neural network training frameworks can be supported through an extensible mechanism.

### 2.3. Federated learning topologies

(Rieke et al 2020) describes the two major different federation topologies, (i) FL using an aggregation server, a hub-and-spoke/‘star’ architecture where collaborating sites share model updates to a central server for combination, and (ii) FL using a peer-to-peer connection, where each collaborator either sends the model updates to each other concurrently (i.e. ‘swarm learning’ Warnat-Herresthal et al 2021) or iteratively (‘Institutional Incremental Learning’ happens when each collaborator passes weights around once, ‘Cyclic Institutional Incremental Learning’ is process of doing the transfer multiple times Sheller et al 2020). For the sake of clarity, this manuscript focuses of FL using an aggregation server.

**Figure 4.**
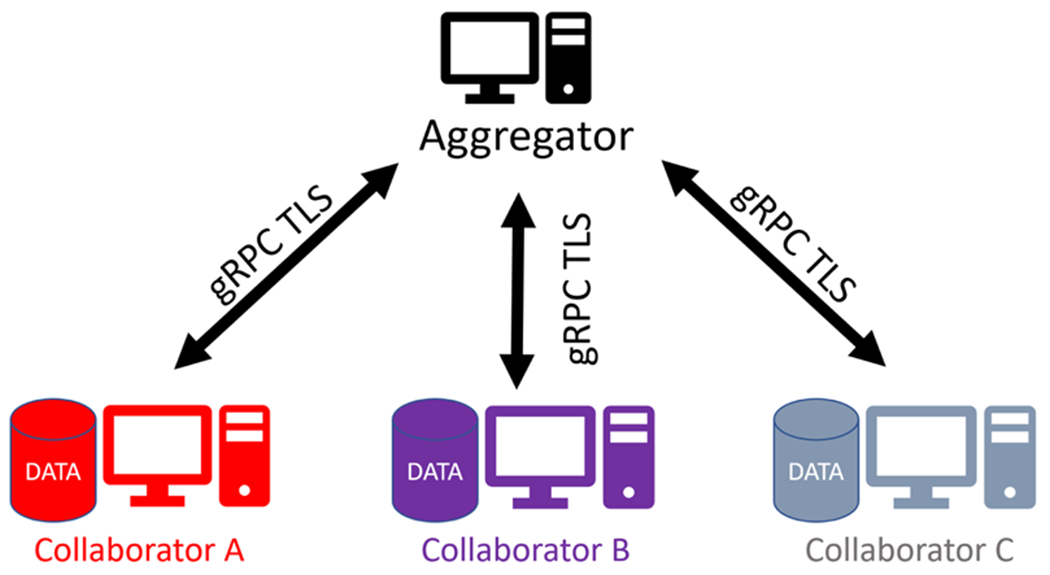
The OpenFL network topology. The federation is a star topology with two types of nodes: collaborators and aggregators. The data of a collaborator remains within that node for local training. The dataset never leaves the collaborator node. Instead, model updates from each collaborator node are sent to an aggregator node so that they can be combined into a global consensus model. The global model is returned to the collaborator nodes for a further round of local training. Collaborators connect with the aggregator through remote procedure calls over mutual TLS connections.

Figure 4 shows the architecture for the OpenFL network topology based on an aggregation server. Each participant in the federation is defined as either a *collaborator* or an *aggregator* node. A collaborator node contains the dataset that is owned by that participant. The hardware of that collaborator node is used to train the ML model locally. The dataset never leaves the collaborator node. An aggregator node is a compute node that is trusted by each collaborator node. Collaborator nodes connect directly to the aggregator node in a star topology. The collaborator nodes connect to the aggregator node through remote procedure calls (gRPC^8^ Wang et al 1993) via a mutual transport layer security (mTLS) (Dierks et al 1999) network connection. Sensitive information such as tasks, model and optimizer weights, and aggregated metrics pass between the collaborator and the aggregator nodes over this encrypted channel.

### 2.4. Security

FL addresses issues of the current paradigm for multi-institutional collaborations based on data pooling, due to its nature to share only model updates across collaborating institutions. However, it introduces new privacy, security, and confidentiality challenges for both AI model developers and data owners/collaborators (Kairouz et al 2019). More specifically, AI model developers may wish to protect their model intellectual properties (IP) as the model gets trained in decentralized environments, while data owners/collaborators would like to ensure that their data cannot be extracted by inspecting the model weights over federated rounds. OpenFL design prioritizes key security concepts such as narrow interfaces, code reuse, open-source code, simplified information security reviews, and code design fit for running on trusted compute hardware, such as a TEE.

#### 2.4.1. PKI certificates

OpenFL uses mutual transport layer security (mTLS) connections^9^ (Dierks and Rescorla 2008). To establish the connection, a valid public key infrastructure certificate (Albarqi et al 2015) signed by a trusted certificate authority, must be provided by all participants. OpenFL provides a method for creating a trusted certificate authority (from within the federation’s collaborating sites), and generating X.509 (Albarqi et al 2015) certificates, but this mechanism is only intended for **non-production testing**, such as academic research. In production environments (for example, when multiple institutions are working together and may not jointly trust an internal CA), it is recommended that an external certificate authority generates the PKI certificates. The minimum recommended certificates are *RSA SHA*-384 3072-*bit* or *ECDSA secp*384*r*. Notably, gRPC connections default to the best ciphersuite available, which is TLS 1.3 with *ECDHE-RSA-AES*256-*GCM-SHA*384.

#### 2.4.2. Trusted execution environments

TEEs offer hardware based memory encryption that isolate specific application code and data in memory and enforces access to it with hardware. For FL, the three key security properties required in a TEE are (1) *confidentiality* of the execution to mitigate attacks such as copying model IP out of memory as the training process executes, (2) *integrity* of the execution to mitigate attacks that alter the behavior of the code, and (3) *remote attestation* of the execution, wherein a TEE can provide some measurements as a proof for the initial execution state to a remote relying party to attest the TEE itself is interacting with the intended code on the intended hardware (Kairouz et al 2019). Recent hardware solutions can provide these three security properties at near native speed, supporting memory (RAM) sizes necessary for training large DL models. Several key OpenFL researchers also worked on Intel Secure Guard Extensions (Intel SGX), and hence OpenFL is natively designed to properly leverage TEEs.

In OpenFL, all applications (including collaborators and aggregator) are executed in a distributed manner for exchanging the ML/DL model information to help improve the training performance. As such, both ML models and distributed data silos need to be protected during training. Leveraging TEE for OpenFL helps protect both model IP and data privacy. Typically, extensive modifications are required to allow the execution of the ML training code inside a TEE that increases development efforts in a user’s application. To address this additional required effort, Gramine (or Gramine SGX) (Tsai et al 2017) is developed as a lightweight, open-source library OS for running unmodified user applications inside Intel SGX, thereby allowing users to run OpenFL code seamlessly without any modifications. We think that this execution of unmodified applications in the enclave will greatly increase the usability for application developers to benefit from security features, such as integrity and confidentiality. Instructions to run OpenFL with Intel SGX using Gramine are outside the scope of this manuscript, and can be found in its documentation^10^.

### 2.5. Running a federation

The training process begins when each collaborator establishes a secure connection to either a central aggregation server (in the case of federated aggregation using a server) or with each other (in case of swarm or cyclic weight transfer). Once the secure connection is established, the initial model weights are passed to the collaborating sites. Collaborating sites can then begin training the same network architecture on their local data for a predefined number of epochs, and share model updates with either each other, or the central aggregation server (depending on the FL topology). Once all the individual submitted updates are combined in the global consensus model, the latter gets sent back to each collaborating site to continue their local training. Each such iteration is called a ‘*federated round*’. The number of federated rounds and epochs to train can be defined in the learning plan.

OpenFL has two methods for developing federations: the Python API and the fx CLI. The CLI is considered the better path for scaling federations within a production environment. The Python API is easier to understand for the data scientist who is working with OpenFL for the first time. Nevertheless, the OpenFL tutorials and demos should allow users to quickly grasp both methods^11^.

## 3. Use cases

### 3.1. The real-world federated tumor segmentation initiative

**Figure 5.**
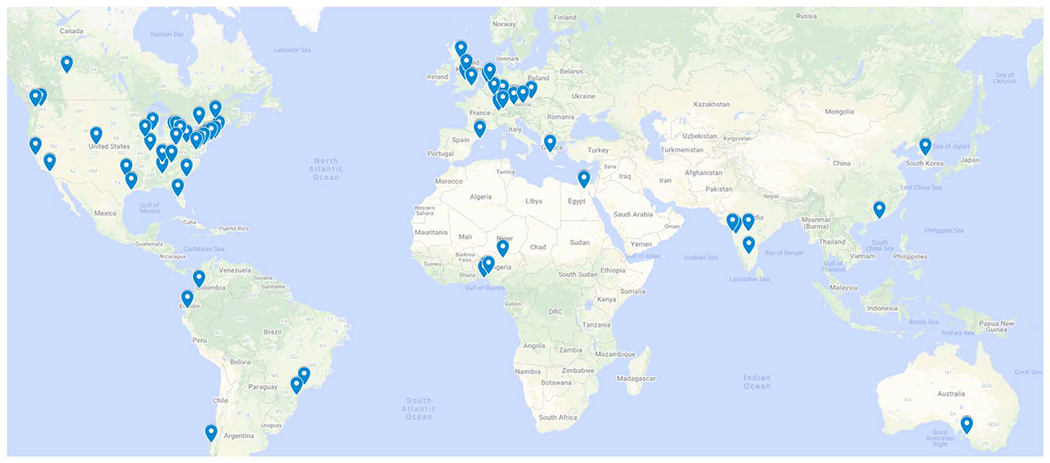
The collaborative network of the first FeTS federation.

The **Fe**derated **T**umor **S**egmentation (FeTS) initiative, led by the University of Pennsylvania, describes an ongoing development of the largest international federation of healthcare institutions aiming at gaining knowledge for tumor boundary detection from ample and diverse patient populations without sharing any patient data (Baid et al 2021, Pati et al 2022a). To facilitate this initiative, a dedicated open-source platform with a user-friendly graphical user interface was developed (Pati et al 2022b). This platform seeks: (i) bringing state of the art pre-trained segmentation models of numerous algorithms (Pati et al 2021b) and label fusion approaches (Pati and Bakas 2021) closer to clinical experts and researchers, thereby enabling easy quantification of new radiologic scans and comparative evaluation of new algorithms, and (ii) allowing multi-institutional collaborations via FL by leveraging OpenFL, to improve these pre-trained models without sharing patient data, thereby overcoming legal, privacy, and data-ownership challenges. FeTS has been initially deployed towards the task of detecting the boundaries of brain tumor sub-compartments, for the most common malignant brain tumor (i.e. glioblastoma) but still a rare disease based on its incidence rates, by utilizing data from *n* = 71 clinical sites spread all around the world (figure 5).

### 3.2. The first computational challenge on federated learning

International challenges have become the *de facto* standard for benchmarking computational analysis methods, including those designed for the healthcare domain. However, the actual performance of even the winning algorithms on ‘real-world’ clinical data often remains unclear, as the data included in these challenges are usually acquired in very controlled settings at few institutions. The seemingly obvious solution of just collecting increasingly more data from more institutions in such challenges does not scale well due to privacy and ownership hurdles.

The first computational challenge ever proposed for FL, was the FeTS 2021 challenge^12^ that focused on benchmarking methods for both the federated training and the federated evaluation of tumor segmentation models (Pati et al 2021a), and was conducted in conjunction with the medical image computing and computer assisted interventions (MICCAI) conference. Specifically, the FeTS 2021 challenge uses clinically acquired, multi-institutional MRI scans from the international brain tumor segmentation (BraTS) 2020 challenge (Menze et al 2014, Bakas et al 2017, 2018), as well as from various remote independent institutions included in the collaborative network of the FeTS real-world federation (section 3.1). The challenge focuses on the construction and evaluation of a consensus model for the segmentation of intrinsically heterogeneous (in appearance, shape, and histology) brain tumors, namely gliomas. Compared to the BraTS challenge, the ultimate goal of the FeTS challenge is divided into the following two tasks:

- **Task 1** (‘Federated training’) aims at effective weight aggregation methods for the creation of a consensus model given a pre-defined segmentation algorithm for training, while also (optionally) accounting for network outages.
- **Task 2** (‘Federated evaluation’) aims at robust segmentation algorithms, given a pre-defined weight aggregation method, evaluated during the testing phase on unseen datasets from various remote independent institutions of the collaborative network of the **fets.ai** federation.

OpenFL enabled innovation on aggregation via a modular API for implementing custom aggregation algorithms.

### 3.3. Predicting acute respiratory distress syndrome & death in COVID-19 patients

Montefiore Health System represents one of the largest integrated care delivery systems in New York. It comprises 11 hospitals, distributed across the poorest (Bronx, NY) and most affluent (Westchester County, NY) communities of the United States.

Early in the COVID-19 pandemic (March–May 2020) the Montefiore Health System used OpenFL to simultaneously assess data from its network hospitals to optimize the sample size necessary to develop, validate, and deploy a clinically reliable DL model (using long short term memory-LSTM models) to predict the likelihood of acute respiratory distress syndrome, as well as death in COVID-19 patients hospitalized in the intensive care unit. The model was deployed and integrated to the routine clinical work flows, in order to provide real-time monitoring, triaging, and clinical decision support to critical care units across the complete health system, by helping identify the highest risk patients (and those with deteriorating health status) hours in advance of an irreversible terminal event.

The results demonstrated validation of the model for accuracy, and timeliness compared to traditional centralized learning, emphasizing the identical learning capabilities and accuracy of the privacy preserving FL, but with additional benefit of agility, more privacy, more confidentiality, more security, scalability, representativeness, and portability of the models to much larger patient populations, and designed to not compromise the privacy of protected health information.

### 3.4. Understanding the physiological effects of radiation exposure on astronauts

Scientists from NASAs Frontier Development Lab (FDL) are using FL to study astronaut health to help better understand the physiological effects of space radiation on humans. Using OpenFL, FDL scientists have created a first-of-its-kind biomarker detection algorithm for cancer that uses data on the effects of radiation on humans and mice. The astronaut health team proved rodent radiation data can be used as a homologue of human radiation data, which is used to train the human algorithm. The causal ML method tackles the researchers scientific challenge to more accurately predict the genes that will be affected by radiation, some relating to cancer and others to immunity response.

This research leveraged OpenFL on Google Cloud Platform, to make it possible to train and combine CRISP 2.0 models (Causal Relation and Inference Search Platform) from institutions such as NASA, Mayo Clinic, and NASAs Gene Lab, without moving/sharing the data to a centralized location. This was crucial because even though each organization had the necessary right to use the data, the data was private and the cost of transmitting data that could be generated aboard a spacecraft was high. With the use of OpenFL, researchers were able to initialize a federated experiment with an ensemble of causal inference methods (represented by a collection of linear and nonlinear invariant risk minimization Arjovsky et al 2019 models) pre-trained on mouse data, select the highest variance human genes and respective mouse homologues across collaborator dataset distributions, and conduct more than 30 rounds of federated training. Finally, CRISP 2.0 was used to output results for further analyses and insights. Using strong overlap in the top-50 features in the federated cross-organism analysis, the study found the previously unidentified gene *SLC*8*A*3 as a potential causal target for further research (O’Donoghue et al 2021).

### 3.5. Highlighted tutorials for potential applications

While some of the first real world use cases that leveraged OpenFL originated in medical imaging and healthcare applications, the underlying framework is designed for broader applicability and to support novel DL research. Because of OpenFL’s built-in support for TensorFlow and PyTorch, OpenFL can easily support higher level DL frameworks and applications, such as those focusing on DL transformers (Wolf et al 2019), keyword spotting (Baevski et al 2020, Yang et al 2021), and synthesis (such as generative adversarial networks). A special use case is that of anomaly detection, which has important applications in industrial cases, where it can be used to robustly and accurately detect defects in the manufacturing process. OpenFL allows federation of a well-known application for this purpose, the PatchSVDD algorithm (Yi and Yoon 2020).

## 4. Discussion

In this manuscript we have introduced the open federated learning (OpenFL, github.com/intel/openfl), an open-source software library for FL. Although OpenFL was originally developed as part of a collaborative project between Intel Labs and the University of Pennsylvania on FL for healthcare, it continues to be developed for general-purpose real-world applications by Intel and the open-source community in GitHub^13^.

Kaushal et al (2020) recommend that researchers need greater access to large and diverse datasets, in order to generate accurate models (Kaushal et al 2020). Without this greater access, they argued, AI models may also have inherent biases and perpetual inequalities. For example, Larrazabal et al (2020) demonstrated that introducing a gender imbalance while training convolutional neural network model to detect disease from chest x-rays resulted in poor performance on the underrepresented gender (Larrazabal et al 2020). This potential for bias is not limited to the healthcare sector. Buolamwini and Gebru (2018) demonstrated that a lack of diversity in training data can lead to significant racial bias in facial detection algorithms. Coston et al (2019) described the harmful effects as a covariate shift in risk models for the financial sector (Coston et al 2019).

FL is an attractive approach to training AI on large, diverse datasets requiring data privacy (Suzumura et al 2019, Rieke et al 2020). Although there is no inherent guarantee that accessing more data translates to accessing better data, it is certainly a step in the right direction toward improving accuracy and reducing bias in AI algorithms. It should be stressed that it is the greater access to data that gives FL an advantage over centralized learning, rather than any inherent algorithmic improvement. Sheller *et al* previously showed that FL can achieve similar accuracy as centralized learning, but may be superior to similar collaborative learning techniques and to training on data from a single institution (Sheller et al 2019, 2020).

The current paradigm for developing DL and ML models in a collaborative healthcare setting currently lacks diversity in data, posing a risk of creating and continuing harmful biases on how algorithms are developed and trained. These gaps can lead to continued health disparities and inequities for underrepresented communities. The National Institutes of Health (NIH) has several programs that aim to increase the availability of data of populations underrepresented in biomedical research. Specifically, the NIH *All of Us*^14^ program aims to recruit participants for underrepresented communities. More than 80% of the core participants represent populations historically underrepresented in biomedical research. NIH’s *All of Us* takes a centralized data approach making longitudinal clinical, genomics, survey, wearable, and survey data available to researchers via the researcher workbench^15^. Recently, the NIH has also taken a federated data approach to address the availability of diverse data and address health disparities. The NIH’s artificial intelligence/machine learning consortium to advance health equity and researcher diversity *AIM-AHEAD* program^16^ will provide federated access to electronic health record data, image data, and social determinants of health. The federated infrastructure will enable researchers develop, and enhance AI/ML algorithms, as well as apply AI/ML approaches to address health inequities and disparities. This direction is designed to encompass more improved healthcare, prevention, diagnoses, and treatments, as well as facilitate intervention and implementation strategies.

The FL concept introduced in this paper is what is implicitly known in the literature as horizontal federated learning (HFL). In these HFL types of federations, each collaborating site collaborates by sharing the knowledge of their local data in the learning process of a global consensus model. While data are different across the collaborating sites, they share both type and format, and are expected to be normalized to fit the same model. However, HFL is not the only way to implement FL pipelines. Vertical federated learning (VFL) is another variant that is quickly rising for its increased flexibility (Wei et al 2022). With VFL the collaborating sites can contribute to a federation by sharing different types of data, vertically partitioned. This means that each collaborating site is providing just a subset of the information required to fit the model. For example, a hospital might want to collaborate with the dentist, pharmacies, and physiotherapist associations, to have a broader clinical understanding of the shared patients. In this case, each collaborating site would only be sharing some features of the final descriptor, implicitly augmenting the security of the paradigm. While OpenFL can support the complex data pre-processing required for VFL, it currently lacks the flexibility to delegate interdependent tasks across federation participants.

From this perspective, VFL represents a limitation of the current version of OpenFL. Another potential limitation is represented by the application scenario. OpenFL can be a good match for FL pipelines among collaborating sites with hub-and-spoke topologies, where having a central aggregation unit does not represent an issue. This might not be the case of a smart environment made of edge devices that work as independent entities connected to the same meshed network, through an all-to-all communication schema. While OpenFL can be deployed through containers, and used with standard libraries and open-source frameworks (e.g., Keras, TensorFlow, PyTorch) that can be deployed to edge devices, it is currently not able to support an all-to-all aggregation mechanism. OpenFL was originally designed for synchronous FL pipelines. However, in large scale deployments, synchronous FL can be problematic because some collaborators may periodically become unresponsive or may take substantially longer to deliver that round’s model contributions due to slower hardware or larger datasets. This problem motivates further exploration into federation that permit asynchronous updates.

As the future outlook for OpenFL, taking into consideration that FL is still a relatively new concept with rapidly evolving developments and advancements, it is our preference to be driven by community requirements that will benefit either research or industrial applications. For example, depending on the attraction that VFL might gain, we intend to extend the current OpenFL capabilities accordingly, with modules needed to implement it. As currently planned immediate future directions, we would like to extend the training beyond DL algorithms, by adding the functionality of running federations based on traditional ML approaches. While the current version of OpenFL is already designed to welcome such changes, it might not be immediately accessible to end-users. Another feature we would like to include concerns fine grained control over tasks that run on specific infrastructures, e.g., the opportunity to have custom aggregator tasks. In addition, enhancing the communication options by opening the FL scenarios to asynchronous updates could enrich OpenFL.

## 5. Conclusion and future outlook

We have introduced the open federated learning (OpenFL, github.com/intel/openfl) library, as a production ready FL package that allows developers to train ML models on the nodes of remote data owners/collaborating sites. The OpenFL interface makes it easy for data scientists to port their existing ML models, whether in TensorFlow, PyTorch, MXNet, or some other ML framework, into a distributed training pipeline. Although OpenFL’s initial use case was in healthcare, the library is designed to be agnostic to the use case, industry, and ML framework, which contributed in being adopted by companies because of its unique focus on security. The development of OpenFL has benefited significantly from its external collaborations, and by making the project open-source we hope that it will continue to be shaped by the wider FL community in new and exciting avenues. Our goal with OpenFL is not to compete with other FL open-source software efforts, but to inter-operate and collaborate towards providing a comprehensive solution for data-private collaborative learning.

Our ambition is that federations, such as the FeTS Initiative^17^, will not serve as *ad hoc* collaborations for specific research efforts, but will serve as permanent collaborative networks for researchers in healthcare and biological research, and also generalize to the financial, industrial, and retail industries to more effectively train, deploy, monitor, and update their AI algorithms over time.

## Footnotes

5
https://pypi.org/project/openfl/

6
https://yaml.org/

7
https://openfl.readthedocs.io/en/latest/running_the_federation.html#aggregator-based-workflow

8
https://grpc.io/

9
https://en.wikipedia.org/wiki/Mutual_authentication

10
https://github.com/intel/openfl/blob/develop/openfl-gramine/MANUAL.md

11
https://openfl.readthedocs.io/en/latest/running_the_federation.notebook.html

12
https://www.med.upenn.edu/cbica/fets/miccai2021/

13
https://github.com/intel/openfl

14
https://allofus.nih.gov/

15
https://www.researchallofus.org/data-tools/workbench/

16
https://datascience.nih.gov/artificial-intelligence/aim-ahead

17
https://www.fets.ai

## References

[R1] Abadi M 2016 Tensorflow: a system for large-scale machine learning 12th USENIX symp. on operating systems design and implementation (OSDI) 16, pp 265–83

[R2] Albarqi A 2015 Public key infrastructure: a survey J. Inf. Secur 6 31–7

[R3] Annas GJ 2003 HIPAA regulations-a new era of medical-record privacy? New Engl. J. Med 348 1486–90 12686707 10.1056/NEJMlim035027

[R4] Arjovsky M 2019 Invariant risk minimization https://doi.org/10.48550arXiv:1907.02893

[R5] Baevski A 2020 wav2vec 2.0:a framework for self-supervised learning of speech representations arXiv:2006.11477

[R6] Baid U 2021 NIMG-32. the federated tumor segmentation (fets) initiative: the first real-world large-scale data-private collaboration focusing on neuro-oncology Neuro-Oncology 23 vi135–vi136

[R7] Baid U 2022 Federated learning for the classification of tumor infiltrating lymphocytes arXiv:2203.16622

[R8] Bakas S 2017 Advancing the cancer genome atlas glioma MRI collections with expert segmentation labels and radiomic features Sci. Data 4 1–13 10.1038/sdata.2017.117 PMC5685212 28872634

[R9] Bakas S 2018 Identifying the best machine learning algorithms for brain tumor segmentation, progression assessment, and overall survival prediction in the BRATS challenge arXiv:1811.02629

[R10] Bonawitz K 2019 Towards federated learning at scale: System design arXiv:1902.01046

[R11] Buolamwini J and Gebru T 2018 Gender shades: intersectional accuracy disparities in commercial gender classification Conference on Fairness, Accountability and Transparency. PMLR pp 77–91 (http://proceedings.mlr.press/v81/buolamwini18a.html)

[R12] Chen T 2015 MXNet: a flexible and efficient machine learning library for heterogeneous distributed systems CoRR arXiv:abs/1512.01274

[R13] Coston A 2019 Fair transfer learning with missing protected attributes Proc. of the 2019 AAAI/ACM Conf. on AI, Ethics, and Society. AIES ’ 19. Honolulu, HI, USA: Association for Computing Machinery 91–8

[R14] Dierks T and Rescorla E 2008 The Transport Layer Security (TLS) protocol version 1.2 The transport layer security (TLS) protocol version 1.2 (https://rfc-editor.org/rfc/rfc5246)

[R15] Dierks T 1999 TLS Protocol Version 1 0–0

[R16] Kairouz P 2019 Advances and open problems in federated learning Foundations and Trends in Machine Learning 14 1–210 Now Publishers, Inc.

[R17] Kaushal A, Altman R and Langlotz C 2020 Health care AI systems are biased Scientific American 11 17 (https://cientificamerican.com/article/health-care-ai-systems-are-biased/)

[R18] Larrazabal AJ 2020 Gender imbalance in medical imaging datasets produces biased classifiers for computer-aided diagnosis Proc. Natl Acad. Sci 117 12592–4 32457147 10.1073/pnas.1919012117 PMC7293650

[R19] Mårtensson G 2020 The reliability of a deep learning model in clinical out-of-distribution MRI data: a multicohort study Med. Image Anal 66 101714 33007638 10.1016/j.media.2020.101714

[R20] McMahan B 2017 Communication-efficient learning of deep networks from decentralized data Artificial Intelligence and Statistics. PMLR pp 1273–82 (http://proceedings.mlr.press/v54/mcmahan17a)

[R21] Menze BH 2014 The multimodal brain tumor image segmentation benchmark (BRATS) IEEE Trans. Med. Imaging 34 1993–2024 25494501 10.1109/TMI.2014.2377694 PMC4833122

[R22] O’Donoghue O 2021 Invariant risk minimisation for cross-organism inference: substituting mouse data for human data in human risk factor discovery arXiv:2111.07348

[R23] Paszke A 2019 Pytorch: An imperative style, high-performance deep learning library Advances in Neural Information Processing Systems pp 8026–37 (10.5555/3454287.3455008)

[R24] Pati S and Bakas S 2021 LabelFusion: medical Image label fusion of segmentations. Version 1.0.10 (10.5281/zenodo.4633206)

[R25] Pati S 2021a The federated tumor segmentation (fets) challenge arXiv:2105.05874 10.1088/1361-6560/ac9449 PMC9592188 36137534

[R26] Pati S 2021b GaNDLF: a generally nuanced deep learning framework for scalable end-to-end clinical workflows in medical imaging arXiv:2103.01006

[R27] Pati S 2022a Federated learning enables big data for rare cancer boundary detection arXiv:2204.10836 10.1038/s41467-022-33407-5 PMC9722782 36470898

[R28] Pati S 2022b The federated tumor segmentation (FeTS) tool: an open-source solution to further solid tumor research Phys Med Biol 67 204002 10.1088/1361-6560/ac9449 PMC9592188 36137534

[R29] Paullada A 2021 Data and its (dis) contents: a survey of dataset development and use in machine learning research Patterns 2 100336 34820643 10.1016/j.patter.2021.100336 PMC8600147

[R30] Rieke N 2020 The future of digital health with federated learning Npj Digit. Med 3 1–7 33015372 10.1038/s41746-020-00323-1 PMC7490367

[R31] Sheller MJ 2019 Multi-institutional deep learning modeling without sharing patient data: a feasibility study on brain tumor segmentation Brainlesion 11383 92–104 31231720 10.1007/978-3-030-11723-8_9 PMC6589345

[R32] Sheller M 2020 Federated learning in medicine: facilitating multi-institutional collaborations without sharing patient data Sci Rep. 10 12598 32724046 10.1038/s41598-020-69250-1 PMC7387485

[R33] Suzumura T 2019 Towards federated graph learning for collaborative financial crimes detection arXiv:1909.12946

[R34] Tsai C-C, Porter DE and Vij M 2017 Graphene-SGX: a practical library os for unmodified applications on SGX 2017 USENIX Annual Technical Conference (USENIX ATC 17). Santa Clara, CA: USENIX Association pp 645–58 (https://usenix.org/conference/atc17/technical-sessions/presentation/tsai)

[R35] Voigt P and Von A 2017 The eu general data protection regulation (gdpr) A Practical Guide vol 10 I edn (Cham: Springer International Publishing) 3152676 (10.5555/3152676)

[R36] Wang X, Zhao H and Zhu J 1993 GRPC: A communication cooperation mechanism in distributed systems ACM SIGOPS Oper. Syst. Rev 27 75–86

[R37] Warnat-Herresthal S 2021 Swarm learning for decentralized and confidential clinical machine learning Nature 594 265–70 34040261 10.1038/s41586-021-03583-3 PMC8189907

[R38] Wei K 2022 Vertical federated learning: challenges, methodologies and experiments arXiv:2202.04309

[R39] Wolf T 2019 Huggingface’s transformers: state-of-the-art natural language processing arXiv:1910.03771

[R40] Yang Q 2019 Federated Machine Learning: concept and Applications ACM Transactions on Intelligent Systems and Technology (TIST) 10 1–19

[R41] Yang S-w 2021 Superb: speech processing universal performance benchmark arXiv:2105.01051

[R42] Yi J and Yoon S 2020 Patch svdd: patch-level svdd for anomaly detection and segmentation Proceedings of the Asian Conference on Computer Vision (Springer) (10.1007/978-3-030-69544-6_23)

[R43] Zech JR 2018 Variable generalization performance of a deep learning model to detect pneumonia in chest radiographs: a cross-sectional study PLoS Med. 15 e1002683 30399157 10.1371/journal.pmed.1002683 PMC6219764

